# Combined Dysfunction of the Amygdala and Nucleus Basalis Underlies Visual Hallucinations in Parkinson's Disease

**DOI:** 10.1002/mds.70011

**Published:** 2025-08-13

**Authors:** Anna Ignatavicius, Lachlan Churchill, Jack Anderson, Ajay Konuri, Claire O'Callaghan, Simon J.G. Lewis, Elie Matar

**Affiliations:** ^1^ Central Clinical School, Faculty of Medicine and Health University of Sydney Sydney New South Wales Australia; ^2^ Parkinson's Disease Research Clinic, Faculty of Medicine Macquarie University Sydney New South Wales Australia; ^3^ Brain and Mind Centre and School of Medical Sciences, Faculty of Medicine and Health University of Sydney Sydney New South Wales Australia; ^4^ Centre for Integrated Research and Understanding of Sleep Woolcock Institute of Medical Research Sydney New South Wales Australia; ^5^ Department of Neurology Royal Prince Alfred Hospital Camperdown New South Wales Australia

**Keywords:** Lewy body, psychosis, acetylcholine, limbic system, fMRI

## Abstract

**Background:**

Visual hallucinations (VHs) are a common feature of Parkinson's disease (PD) believed to arise from disruptions to the functional architecture supporting sensory integration and attentional control. Across synucleinopathies, increased pathological burden in the amygdala and deficits in cholinergic modulation have been linked to VHs. However, the interaction of these changes and their combined contribution to the neurobiological mechanisms underlying hallucinatory phenomena remain poorly understood.

**Objectives:**

To investigate the convergent impact of amygdala and cholinergic dysfunction on VHs in PD.

**Methods:**

Seventy patients with PD, including 30 with and 40 without VHs, underwent structural and resting‐state functional magnetic resonance imaging. Seed‐based analyses were performed to examine whether altered functional connectivity between the bilateral amygdala and the cholinergic nucleus basalis of Meynert (NBM) with cortical networks involved in attention and visual processing is related to the presence of VHs.

**Results:**

Patients with VHs exhibited reduced amygdala connectivity with the visual network and reduced left amygdala connectivity with both dorsal and ventral attentional networks compared with those without VHs. Furthermore, mediation analyses indicated that the association between amygdala‐attentional network dysconnectivity and VHs was at least partially explained by functional interactions between the left NBM and the ventral attention network. These functional alterations were not associated with amygdala or NBM volumes, suggesting they may occur independently of measurable gray matter atrophy.

**Conclusions:**

Our findings demonstrate that VHs are associated with a network signature of impaired functional connectivity linking the amygdala, cholinergic dysfunction, and cortical networks associated with attention and perception. These results highlight the interplay between distinct but related neural circuitries and provide new insights into the pathophysiological mechanisms of VHs in PD. © 2025 The Author(s). *Movement Disorders* published by Wiley Periodicals LLC on behalf of International Parkinson and Movement Disorder Society.

Visual hallucinations (VHs) are a common nonmotor feature of Parkinson's disease (PD), ranging from minor misperceptions and illusions to vivid, lifelike images that are typically emotionally neutral but can become distressing as insight fades.^1^ These symptoms increase in prevalence with disease progression and have been associated with a more aggressive clinical trajectory,^2^, ^3^ emphasizing the crucial need for a more detailed understanding of their underlying neurobiological mechanisms.

The phenomenological symptoms of VHs have been conceptualized as a consequence of an imbalance between top‐down and bottom‐up perceptual processing. From a systems‐level perspective, impaired coordination between attentional and salience brain networks, driven by neurotransmitter dysfunction and localized pathology, leads to an overreliance on internally generated predictions in an attempt to resolve noisy visual input.^4^, ^5^, ^6^, ^7^, ^8^ Within this framework, the default mode network (DMN), which is associated with mental imagery and memory retrieval, exerts excessive influence over perception because of a failure to engage attentional control networks such as the frontoparietal (FPN) and dorsal attention network (DAN).^8^, ^9^ Concurrently, aberrant signaling from the ventral attention network (VAN), which is typically activated by unexpected and potentially relevant stimuli, leads to inappropriate shifts between endogenous and exogenous processing.^8^ Functional magnetic resonance imaging (fMRI) investigations have provided support for this hypothesis, demonstrating that PD patients with VHs (PD‐VHs) exhibit abnormal coupling of the DMN with the visual network, as well as decreased connectivity between the DAN, VAN, and DMN.^10^, ^11^, ^12^ Although the results of these studies have provided valuable insights into the neurobiological correlates of VHs, precisely how alterations within specific cortical‐subcortical circuitries contribute to these dysfunctional network interactions has yet to be fully elucidated.

Considerable evidence suggests that degeneration within the cholinergic system plays a central role in the pathological mechanisms underlying VHs in PD.^13^, ^14^ The nucleus basalis of Meynert (NBM) is the largest of the basal forebrain nuclei and provides the majority of cholinergic input to the neocortex and limbic system reflecting its capacity to orchestrate information processing across the brain.^15^, ^16^, ^17^ Neuroimaging studies have shown widespread cortical cholinergic denervation in PD,^18^, ^19^, ^20^, ^21^ with more severe alterations in regions overlapping the visual and attentional networks in patients who experience VHs.^22^, ^23^ Similarly, VHs in Lewy body dementia, which includes both dementia with Lewy bodies and PD dementia (PDD), have been linked to a reduction in the number of white matter pathways from the NBM to the cortex, along with a loss of structural‐functional coupling.^24^ Together, these findings suggest that cholinergic dysfunction may be implicated in the functional connectivity abnormalities associated with VHs in PD.

In addition to extensive cholinergic deficits, VHs in PD have been consistently associated with an elevated Lewy body burden within the amygdala.^25^, ^26^, ^27^, ^28^ These findings have been corroborated in vivo, with a recent meta‐analysis reporting lower amygdala volumes in PD‐VHs, independent of cognition and disease severity.^29^ The amygdala shares connections with the regions within the ventral visual stream, as well as exogenous and endogenous attentional networks, suggesting it acts as a convergence point for integrating top‐down and bottom‐up information.^30^, ^31^ Indeed, a recently proposed amygdala‐centric model of PD‐related visual dysfunction highlights structural and functional abnormalities within this region as key contributors to impaired visual and attentional processing.^32^ Moreover, the amygdala is a highly vulnerable site of neurodegenerative change and is crucial in current neuropathological staging schemes that emphasize a limbic‐predominant trajectory of the spread of Lewy body pathology.^33^, ^34^ Therefore, exploring how functional disruptions in this nuclear complex correlate with important disease‐related milestones, such as VHs, is also relevant for understanding the heterogeneity of symptom progression in Lewy body disorders.

Despite the compelling evidence implicating these subcortical regions, the combined functional consequences of such pathological alterations to VHs remain poorly understood. The earlier data suggest that the disrupted modulatory influence from the amygdala and the NBM on their respective cortical targets may play a role in the etiology of VHs in PD in distinct yet complementary ways. As a putative subcortical node within the VAN, the amygdala likely exerts its influence on VHs through attentional systems.^8^, ^35^, ^36^ In addition, cholinergic transients are believed to mediate the switch from internal and external processing,^37^, ^38^ potentially operating through the NBM's interactions with core regions of the VAN.^39^, ^40^, ^41^, ^42^ Therefore, the interplay between aberrant cholinergic signaling and amygdala dysfunction may drive VHs in PD by destabilizing the flexible coordination of attentional resources throughout the brain.

In this study, we sought to investigate the potential mechanisms by which the amygdala and NBM jointly contribute to network dysfunction associated with VHs in PD. We hypothesized that PD‐VHs would exhibit altered resting‐state functional connectivity of the amygdala and NBM with visual and attentional networks compared with those without VHs. Furthermore, to evaluate the convergent impact of these subcortical regions on network dysfunction, we performed mediation analyses to determine whether NBM connectivity with the VAN influences the relationship between amygdala‐attentional network connectivity and the presence of VHs. Recognizing that functional alterations can occur independently of structural deterioration,^43^, ^44^ we further explored whether differences in functional connectivity between hallucinating and nonhallucinating PD patients are accompanied by, or associated with, greater atrophy in the amygdala and NBM.

## Patients and Methods

### Participants

Seventy individuals with idiopathic PD, including 30 with VHs (PD‐VHs) and 40 without VHs (PD‐NoVHs), were recruited from the Parkinson's Disease Research Clinic (Sydney, Australia). All patients satisfied the UK Parkinson's Disease Society Brain Bank criteria for PD and displayed no overt signs of dementia.^45^ The study was approved by the local Ethics Committee, and all participants provided written informed consent before assessment, in accordance with the Declaration of Helsinki.

### Clinical and Neuropsychological Testing

Stage of illness and motor‐symptom severity were assessed with the Hoehn and Yahr Scale and the Movement Disorder Society–revised Unified Parkinson's Disease Rating Scale (MDS‐UPDRS) Part III, respectively.^46^ The presence of hallucinations was based on a score ≥ 1 on question 2 in Part I of the MDS‐UPDRS and confirmed with clinical notes to ensure these experiences were consistent with visual hallucinatory phenomena. Further details on psychosis‐related symptoms were collected using questions 1 to 4 of the Scales for Outcomes in Parkinson's disease‐Psychiatric Complications.^47^ The degree of affective symptom severity was determined with the Hospital Anxiety and Depression Scale (HADS),^48^ and the Rapid Eye Movement Sleep Behavior Disorder Screening Questionnaire (RBDSQ) was used to assess sleep disturbances.^49^ The Mini Mental State Examination (MMSE) and Montreal Cognitive Assessment (MoCA)^50^ were used as measures of global cognition. Both assessment and MRI acquisition were performed while patients were on their regular antiparkinsonian medication, with the dopaminergic dose equivalent score calculated according to a standard formula (mg/day).^51^

### MRI Acquisition and Preprocessing

All participants underwent a whole‐brain structural T1‐weighted MRI scan and resting‐state BOLD functional scan within a 6‐month period of their clinical assessment. Imaging data were obtained on a 3‐Tesla MRI scanner (General Electric). Sagittal 3D T1‐weighted images were acquired with an echo time (TE) = 2.7 ms, repetition time (TR) = 7.2 ms, an acquisition matrix of 256 × 256, 200 slices, and a slice thickness = 1 mm. The acquisition parameters for the T2*‐weighted echo‐planar functional scans included: TE = 36 ms, TR = 3000 ms, flip angle = 90°, 32 contiguous axial slices covering the whole brain, field of view = 220 mm, slice thickness = 3 mm, and raw voxel size = 3.75 × 3.75 × 3 mm. The total duration of the resting‐state scan was approximately 7 minutes (140 TRs), and participants were instructed to lie awake with their eyes closed.

Preprocessing of fMRI data was performed using fMRIprep 21.0.2,^52^ followed by a customized denoising strategy using *fMRIDenoise* (https://github.com/compneuro-ncu/fmridenoise), as previously described.^53^ T1‐weighted images were processed using the CAT12 version 12.9^54^ in conjunction with SPM12 (Wellcome Centre for Human Neuroimaging, London, UK; https://www.fil.ion.ucl.ac.uk/spm) implemented in MATLAB R2023a (MathWorks, Natick, Massachusetts, USA). Comprehensive descriptions of each processing step are provided in the Supporting Information.

### Seed‐Based Functional Connectivity Analysis

The bilateral amygdala and NBM were selected a priori as seeds of interest. Both left and right seeds were analyzed separately, because previous studies have indicated potential hemispheric structural and functional asymmetries in these regions.^22^, ^55^, ^56^

The mean BOLD signal time‐series were extracted from resting‐state fMRI data using 400 cortical regions from the Schaefer parcellation,^57^ 54 subcortical regions from the Tian parcellation,^58^ and a probabilistic anatomical map of the NBM derived from microscopic delineations of 10 postmortem human brains.^59^ Functional connectivity matrices were computed for each participant by calculating the pairwise Pearson correlation coefficients between the time‐series of all regions, followed by Fisher's z‐transformation.

The 400 cortical parcels were assigned to one of seven canonical functional networks,^60^ five of which were selected because of their relevance to current models of VHs: the visual network, DAN, VAN, DMN, and FPN. Seed‐network connectivity was calculated as the average connectivity between the seed region and all cortical parcels within each network. Functional connectivity between the amygdala and NBM was also examined to determine whether disrupted coupling between these regions is associated with VHs.

### Gray Matter Volumetric Analysis

Gray matter volumes for the amygdala and NBM were extracted in the subject's native space using the standard CAT12 pipeline. The amygdala and NBM were delineated using the Tian subcortical atlas^58^ and the Julich‐Brain cytoarchitectonic atlas,^59^, ^61^ respectively. These regions of interest were based on the same anatomical maps used to specify seeds in the functional connectivity analysis, ensuring alignment between structural and functional measures. Total amygdala volumes for each hemisphere were determined by summing the estimated gray matter volumes of the lateral and medial subdivisions. Amygdala and NBM volumes were adjusted for estimated total intracranial volume using the residual correction method.^62^

### Statistical Analysis

All statistical analyses were performed using MATLAB R2023a. Group differences were assessed with nonparametric permutation tests (10,000 permutations), whereas differences in sex distribution and antidepressant use between groups were evaluated using χ^2^ tests. Missing quantitative cognitive data (Supporting Information Table S1) were handled using Multivariate Imputation by Chained Equations (MICE) predictive mean matching via the *mice* package in R version 4.3.1.^63^ Functional connectivity and gray matter volume comparisons between groups included age and sex as covariates. The influence of potential confounding factors, such as head motion and demographic variables that differed between groups, was assessed using Spearman rank correlation analyses. Multiple comparisons were corrected for using the Benjamini‐Hochberg procedure to control for the false discovery rate (FDR) with *q* < 0.05.

Mediation analyses were performed to explore whether NBM functional connectivity mediated the association between amygdala‐attentional network functional connectivity and the likelihood of a patient being classified as a hallucinator. Mediation models incorporated both linear and logistic regression to account for continuous and binary outcomes, with age and sex included as covariates of no interest. The indirect effect was assessed using bootstrapping with 10,000 resamples to estimate confidence intervals (CIs) and statistical significance (*P* < 0.05). Further details regarding mediation analysis are provided in the Supporting Information.

## Results

### Demographics

Both PD‐VH and PD‐NoVH groups were well matched for demographic and disease‐related variables. No significant differences were observed in global cognition scores between patient groups. However, PD‐VH demonstrated a higher burden of self‐reported depression and anxiety symptoms, as well as RBD‐related sleep disturbances. Three patients in the PD‐NoVH group and five patients in the PD‐VH group were on antidepressants at the time of assessment. No patients were taking cholinesterase inhibitors or antipsychotic medication. Detailed results from group comparisons of demographic and clinical characteristics are reported in Table 1.

**TABLE 1:** Demographics and clinical profile of Parkinson's disease patients with and without hallucinations

	PD‐VHs (n = 30)		PD‐NoVHs (n = 40)		*P* value
	n	Mean (SD)	n	Mean (SD)	
Sex, M:F (n)	19:11	–	32:8	–	0.175
Age (y)	–	65.5 (6.20)	–	65.2 (10.1)	0.874
Years of education	–	13.6 (3.37)	–	14.7 (3.31)	0.176
TIV (mL)	–	1523 (152)	–	1589 (135)	0.066
Disease duration (y)	–	6.13 (4.70)	–	5.37 (3.44)	0.456
Hoehn & Yahr	–	2.10 (0.55)	–	1.99 (0.56)	0.487
MDS‐UPDRS III	–	29.9 (15.9)	–	25.9 (14.0)	0.270
DDE (mg/day)	–	633 (554)	–	658 (480)	0.854
On antidepressants (total) (n)	5	–	3	–	0.275
SSRI	3	–	1	–	–
SNRI	–	–	1	–	–
TCA	2	–	–	–	–
Atypical	–	–	1	–	–
MMSE	–	28.3 (1.93)	–	28.8 (1.68)	0.244
MoCA	–	26.5 (3.19)	–	27.6 (2.15)	0.080
RBDSQ	–	**6.17 (3.99)**	–	**4.08 (2.81)**	**0.016**
HADS‐Anxiety	–	**5.00 (3.76)**	–	**2.53 (2.39)**	**0.002**
HADS‐Depression	–	**4.93 (3.30)**	–	**2.50 (2.34)**	**0.002**
SCOPA‐PC 1–4	–	**1.90 (2.10)**	–	**0.425 (0.68)**	**<0.001**
MDS‐UPDRS I.2	–	1.37 (0.67)	–	–	–

Question 2 within Part I of the MDS‐UPDRS was used to classify patients as hallucinators (score ≥ 1). Significant differences (*P* < 0.05) between groups are highlighted in bold.

Abbreviations: PD‐VHs, Parkinson's disease patients with visual hallucinations; PD‐NoVHs, Parkinson's disease patients without visual hallucinations; SD, standard deviation; M, male; F, female; TIV, total intracranial volume; MDS‐UPDRS III, Movement Disorder Society–revised Unified Parkinson's Disease Rating Scale Part III; DDE, dopaminergic dose equivalent; SSRI, selective serotonin reuptake inhibitor; SNRI, serotonin‐norepinephrine reuptake inhibitor; TCA, tricyclic antidepressant; MMSE, Mini Mental State Examination; MoCA, Montreal Cognitive Assessment; RBDSQ, Rapid Eye Movement Sleep Behavior Disorder Screening Questionnaire; HADS, Hospital and Anxiety Depression Scale; SCOPA‐PC, Scales for Outcomes in Parkinson's disease‐Psychiatric Complications (questions 1–4).

### Amygdala and Nucleus Basalis Functional Connectivity

**FIG. 1:**
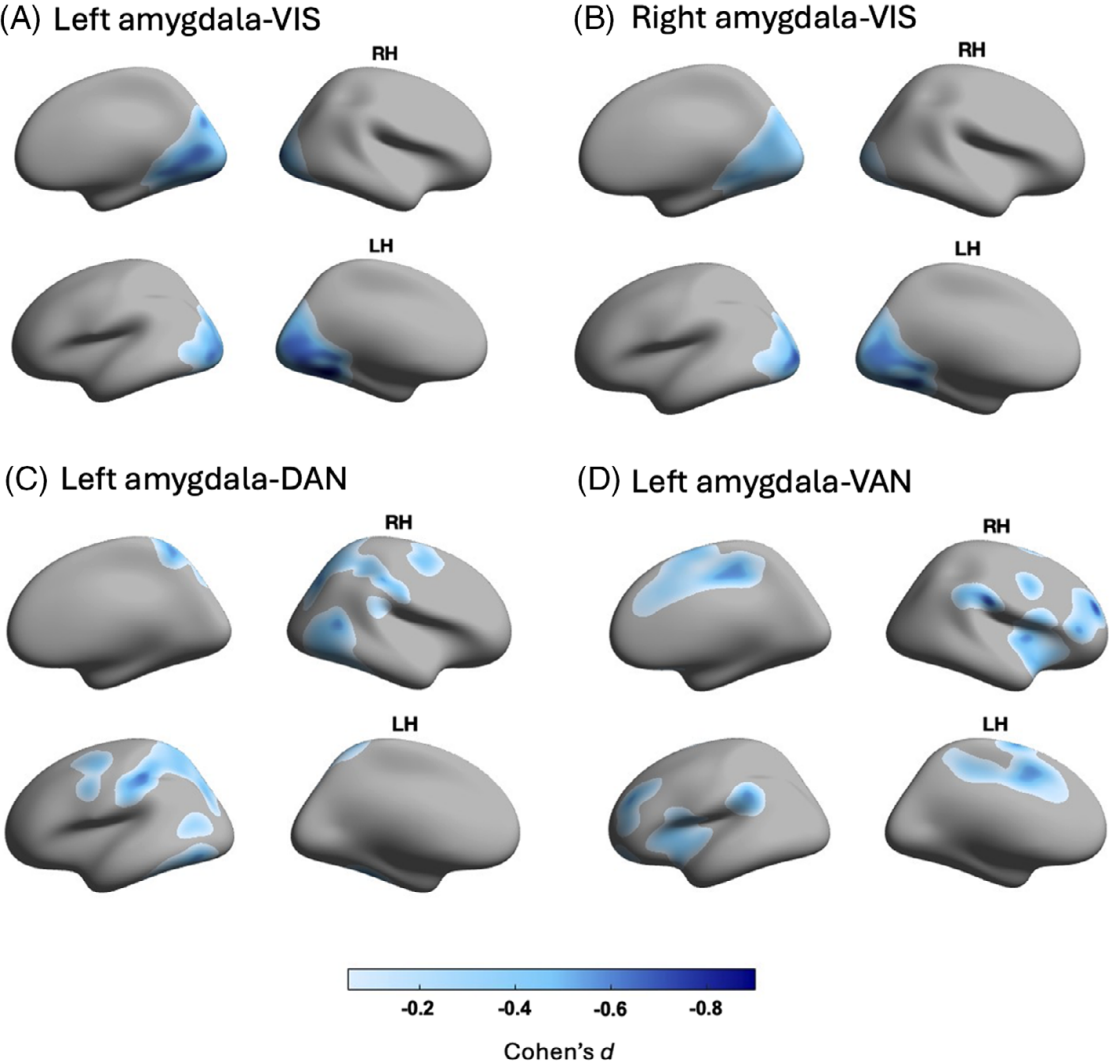
Reduced functional connectivity between the amygdala and cortical networks in Parkinson's disease patients with visual hallucinations (VHs; PD‐VH) compared with those without VHs (PD‐NoVH). (**A**–**D**) Group differences in amygdala‐network functional connectivity between the hallucinating and nonhallucinating groups projected onto lateral and medial views of the right (RH) and left hemispheres (LH). The color scale represents the effect size of the group differences (Cohen's *d*), with darker shades reflecting larger regional reductions in connectivity in PD‐VHs compared with PD‐NoVHs. Only voxels within the masks of the respective cortical networks are shown. (**A**) Left amygdala and the visual network (VIS); (**B**) right amygdala and the visual network (VIS); (**C**) left amygdala and the dorsal attention network (DAN); (**D**) left amygdala and the ventral attention network (VAN). [Color figure can be viewed at wileyonlinelibrary.com]

PD‐VHs exhibited significantly reduced resting‐state functional connectivity between the amygdala bilaterally with the visual network (left: *P*_FDR_ = 0.012, Cohen's *d* = −0.815; Fig. 1A; right: *P*_FDR_ = 0.048, Cohen's *d* = −0.589; Fig. 1B) compared with PD‐NoVHs. The strongest effects were localized to the calcarine sulcus and extrastriate cortex, areas particularly associated with the ventral visual stream. In addition, PD‐VHs showed reduced left amygdala functional connectivity with both the DAN (*P*_FDR_ = 0.032, Cohen's *d* = −0.761; Fig. 1C) and the VAN (*P*_FDR_ = 0.032, Cohen's *d* = −0.789; Fig. 1D) relative to PD‐NoVHs. For the DAN, this was mainly driven by lower amygdala connectivity with the regions within the superior parietal lobule, postcentral gyrus, and the temporo‐occipital cortex. Within the VAN, reduced amygdala connectivity was most pronounced with regions within the lateral prefrontal, frontomedial, and insula cortices. A detailed report of *P* values and effect sizes at the regional level is provided in Supporting Information Table S2.

Group‐level comparisons showed a trend toward reduced functional connectivity between the left NBM and the VAN in PD‐VHs compared with PD‐NoVHs (*P* = 0.018 uncorrected, Cohen's *d* = −0.661); however, this did not survive FDR correction (*P*_FDR_ = 0.060). No significant group differences were observed in functional connectivity between the NBM and other resting‐state networks (*P* > 0.05). Both PD‐VHs and PD‐NoVHs showed positive coupling of the NBM and amygdala, consistent with previous work indicating these regions are functionally connected,^64^ although no significant group differences were observed.

A sensitivity analysis controlling for HADS scores and antidepressant use showed largely consistent results; however, reduced right amygdala connectivity with the visual network no longer survived FDR correction (Supporting Information Table S3).

### Mediating Role of Nucleus Basalis Functional Connectivity

**FIG. 2:**
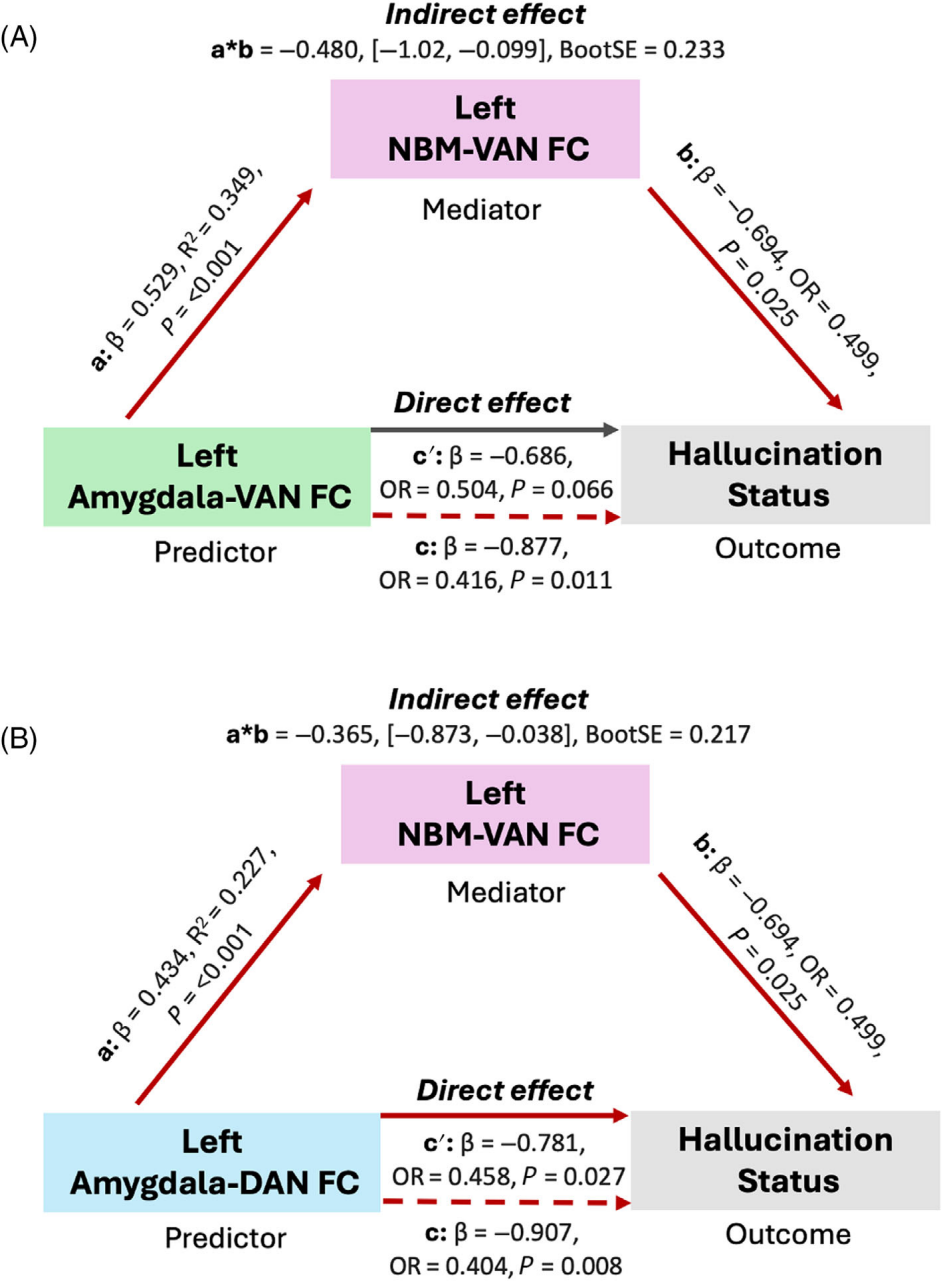
Path diagrams of the mediation models. (**A**) Decreased functional connectivity (FC) between the left nucleus basalis of Meynert (NBM) and the ventral attentional network (VAN) significantly mediated the relationship between reduced left amygdala‐VAN FC and the presence of visual hallucinations (VHs). (**B**) Decreased left NBM‐VAN FC partially mediated the relationship between reduced left amygdala FC with the dorsal attentional network (DAN) and the presence of VHs. Path *a* represents the association between left amygdala‐DAN/VAN FC and left NBM‐VAN FC. Path *b* reflects the relationship between left NBM‐VAN FC and hallucination status. Path *c* shows the total association between left amygdala‐DAN/VAN FC and hallucination status, while path *c′* represents the direct effect when controlling for left NBM‐VAN FC. The indirect effect, which quantifies the mediating role of left NBM‐VAN FC, is derived from the product of paths *a* and *b* and assessed using bootstrapping with 10,000 resamples (95% confidence intervals). BootSE refers to the bootstrap standard error of the indirect effect. The indirect effect is considered to fully mediate the total effect when the direct effect is no longer significant. Conversely, the indirect effect only partially mediates the total effect if the direct effect remains significant. Red arrows indicate significant associations (*P* < 0.05), and all regression weights are fully standardized. OR, odds ratio. [Color figure can be viewed at wileyonlinelibrary.com]

Building on the preceding seed‐network findings, we conducted exploratory mediation analyses to assess whether the relationship between amygdala‐attentional network dysfunction and the presence of VH is dependent on alterations in NBM connectivity with the VAN. Given the trend‐level group difference in left NBM‐VAN connectivity and the well‐established role of the cholinergic system in attentional processes,^65^, ^66^ we included this pathway as a potential mediator. Two separate mediation models were tested: one examining left amygdala‐VAN connectivity (Fig. 2A) and another examining left amygdala‐DAN connectivity (Fig. 2B).

Reduced left amygdala connectivity with both attentional networks significantly predicted lower left NBM‐VAN connectivity (amygdala‐VAN: β = 0.580, *R* ^2^ = 0.349, *P* < 0.001; amygdala‐DAN: β = 0.434, *R* ^2^ = 0.227, *P* < 0.001). In addition, lower left NBM‐VAN connectivity significantly predicted the presence of hallucinations (β = −0.694, odds ratio [OR] = 0.499, *P* = 0.025). Significant indirect effects were observed in both models (amygdala‐VAN: ab = −0.480, 95% CI: [−1.02, −0.099]; amygdala‐DAN: ab = −0.365, 95% CI: [−0.873, −0.038]), indicating that NBM‐VAN connectivity mediated the relationship between amygdala connectivity and hallucination status. The relationship between amygdala‐VAN connectivity and VHs was attenuated and no longer significant after accounting for NBM‐VAN connectivity (*P* = 0.066), consistent with a pattern of full mediation. In contrast, NBM‐VAN connectivity partially mediated the relationship between amygdala‐DAN connectivity and hallucination status, with the direct effect remaining significant when controlling for NBM‐VAN connectivity (*P* = 0.027).

### Additional Control Analyses

Because motion can introduce spurious correlations in resting‐state functional connectivity,^67^ we performed a group comparison of mean framewise displacement, a measure that provides an index of head movement across consecutive volumes. There were no significant differences in framewise displacement between patient groups (*P* = 0.678). Furthermore, none of the significant functional connectivity outcome measures correlated with participants' head movement. No significant correlations were identified between MoCA, HADS, or RBDSQ scores and the functional connectivity measures that differed between PD‐VHs and PD‐NoVHs (all *P* > 0.05; Supporting Information Table S4).

### Comparison of Amygdala and Nucleus Basalis Volumes

No significant group differences in estimated gray matter volume were observed for the amygdala or NBM in either hemisphere (*P* > 0.05; Supporting Information Table S5). Moreover, we found no significant correlation between seed‐network mean functional connectivity and the volumes of the amygdala or NBM (all *P* > 0.05).

## Discussion

**FIG. 3:**
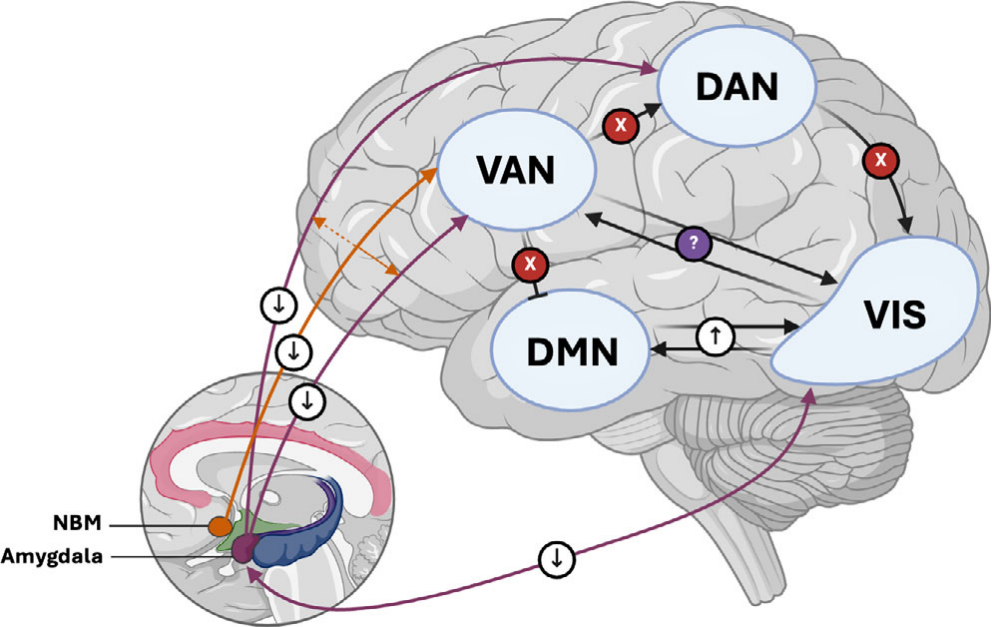
Schematic overview of altered network interactions associated with Parkinson's disease (PD) visual hallucinations (VHs; PD‐VHs). The genesis of VHs in PD has been linked to the disrupted coordination of intrinsic brain networks that subserve attentional and perceptual processing. This figure summarizes previously reported internetwork functional connectivity abnormalities combined with results from this study. The dorsal (DAN) and ventral (VAN) attention networks, default mode network (DMN), and visual network (VIS) are shown on the lateral view of the left hemisphere, while the nucleus basalis of Meynert (NBM) and amygdala appear in the inset. Red “X” icons indicate disrupted connectivity between networks; downward arrows denote decreased connectivity; upward arrows indicate increased connectivity; and the question mark (“?”) signifies inconsistent or variable findings across studies. Prior functional magnetic resonance imaging (fMRI) investigations in PD‐VHs have reported decreased DAN‐VIS connectivity^11^ and increased DMN‐VIS coupling,^11^, ^104^, ^105^, ^106^ along with reduced VAN‐DMN^10^, ^107^ and VAN‐DAN connectivity.^10^, ^108^ Findings on the VAN‐VIS relationship in PD‐VHs are conflicting with some suggesting decreased VIS‐VAN connectivity^108^ and others reporting increased occipital functional connectivity with regions associated with the VAN.^106^, ^107^ Our findings showed hypoconnectivity between the amygdala and both DAN and VAN, as well as the VIS, in PD‐VHs. Reduced NBM‐VAN connectivity mediated the relationship between amygdala‐attentional network connectivity and VHs, with the solid arrow depicting full mediation and the dashed arrow illustrating partial mediation. Overall, these findings emphasize the complex interplay between distributed cortical networks and the modulating influence of subcortical structures, such as the NBM and amygdala, providing further insight into the pathophysiological mechanisms underlying PD‐VHs. [Color figure can be viewed at wileyonlinelibrary.com]

In this study, we examined resting‐state functional connectivity of the amygdala and NBM to address how these regions might participate in the impaired coordination of large‐scale brain networks associated with VHs in PD. In this study, we demonstrate that PD‐VHs have weakened connectivity between the amygdala and key networks supporting visual and attentional processing. Furthermore, we provide novel evidence that the relationship between amygdala‐attentional network connectivity and the likelihood of experiencing VH is mediated by the integrity of functional interactions between the NBM and the VAN. Figure 3 provides a graphical overview of these findings, integrating the present results with prior reports of altered internetwork functional connectivity in PD‐VH. Notably, the differences in functional connectivity observed in hallucinating compared with nonhallucinating patients were independent of amygdala and NBM volumes. This suggests that functional alterations are not driven by significant atrophy in these regions and emphasizes the utility of functional connectivity analyses in uncovering network‐level dysfunction that extends beyond overt structural changes.

The amygdala has long been recognized as a critical hub in the neural circuitry of emotion and fear conditioning.^68^, ^69^ However, there is growing appreciation of its broader modulatory influence across distributed neural networks supporting perception, prioritizing information processing based on the salience, significance, ambiguity, and unpredictability of stimuli.^70^, ^71^, ^72^, ^73^ In PD‐VHs, we observed reduced functional connectivity between the amygdala and the visual network, particularly with regions within the ventral extrastriate areas, compared with PD‐NoVHs. This aligns with previous neuroimaging studies that have linked VHs to altered connectivity and hypometabolism within the occipitotemporal cortex,^74^, ^75^, ^76^, ^77^ and supports recent findings implicating amygdala dysconnectivity with the visual network as an early substrate of visual dysfunction in PD.^32^ The basolateral amygdala projects topographically across the ventral visual stream, including to the primary visual areas, while receiving afferent input from the anterior inferotemporal cortex.^78^, ^79^ These feedback projections are thought to provide reentrant bias signals that modulate ongoing perceptual processing, enhancing responses in both the higher‐order association areas and early visual cortex.^80^, ^81^ Therefore, impaired amygdala signaling within the visual network may reflect a possible pathway relevant to VHs in PD that disrupts ongoing visual processing.

Our findings build on theoretical models of VHs emphasizing a confluence of visual processing impairments and deficits in attentional control,^5^, ^6^, ^8^ suggesting that both cholinergic and amygdala dysfunction may represent core neurobiological substrates underlying the functional mechanisms of VHs in PD. Beyond the visual network, we observed reduced functional connectivity between the left amygdala and both the DAN and VAN in PD‐VHs compared with PD‐NoVHs. The amygdala, particularly its basolateral aspect that is disproportionately affected by Lewy body pathology in PD‐VHs,^25^ is considered a critical node within the VAN.^35^, ^36^ Salient or ambiguous sensory stimuli from the environment engage the basolateral amygdala, which transmits this information to regions within the VAN such as the dorsal anterior cingulate and anterior insula leading to the rapid recruitment of the DAN.^36^, ^82^ The amygdala is also thought to interact independently with the DAN to guide top‐down selective attention to contextually relevant features based on their learned associations.^30^, ^83^, ^84^ Dysfunctional communication between the amygdala and these networks may impair the allocation of attentional resources required for the detection and interpretation of visual input in complex environments. Indeed, during experimentally induced misperceptions, PD‐VHs show a relative inability to engage the DAN, which predicts stronger DMN‐visual network coupling.^11^ Although our findings do not directly assess these dynamic perceptual states, they support prior work suggesting dysfunctional attentional network interactions may contribute to a shift toward internally driven processing in the context of ambiguous or degraded sensory input.^8^

Although no group differences were observed in functional coupling between the NBM and amygdala, reduced amygdala connectivity with the DAN and VAN was more strongly associated with the presence of VHs when accompanied by NBM dysconnectivity. Specifically, the relationship between disrupted amygdala‐VAN functional connectivity and VHs was significantly mediated by impaired interactions between the NBM and VAN. This suggests that, although altered functional connectivity between the amygdala and VAN may be associated with the trait of VHs, it appears to be influenced by concurrent disturbances in cholinergic signaling. In contrast, the relationship between amygdala‐DAN functional connectivity and VHs was only partially mediated by NBM functional connectivity, indicating that amygdala dysfunction may be more directly associated with disrupted top‐down attentional processes relevant to VH, with these effects further amplified within the context of reduced NBM‐VAN coupling. These findings may help reconcile conflicting neuropathological evidence regarding the strength of the association between amygdala pathology and VHs in patients with and without dementia.^25^, ^26^ Our results suggest that amygdala contributions to VHs may emerge when the cholinergic system is also compromised. Altered cholinergic innervation can occur early in a subset of patients with PD,^85^ with more widespread cholinergic deficits associated with the progression to PDD.^86^ This may account for the stronger pathological associations between the amygdala and VHs observed in cases with concomitant dementia.

The ventral frontal and cingulo‐opercular regions of the VAN are densely innervated by highly arborized NBM cholinergic neurons,^41^ which appear to be particularly vulnerable to degeneration in PD.^19^ A recent resting‐state fMRI study found that Lewy body dementia patients with VHs show reduced functional connectivity within the VAN.^87^ Although this study did not specifically attribute these changes to cholinergic dysfunction, evidence indicates that cholinergic neurotransmission plays a pivotal role in shaping cortical network organization and attentional processes, including those underpinned by the VAN.^37^, ^42^, ^65^ Consequently, cholinergic deficits could distort the function of the VAN, which in conjunction with impaired visual processing may compromise precision afforded to bottom‐up sensory information leading to an overweighting of internally generated predictions.^8^, ^88^ Together, our results suggest that the impairment in effective switching between endogenous and exogenous processing hypothesized to underlie VHs may depend, in part, on the functional interactions of the NBM and amygdala with salience and attentional networks.

In view of the earlier network changes, the lack of relationship with atrophy in the amygdala highlights the importance of functional alterations when considering mechanisms underlying paroxysmal phenomena like VHs. Conventional static functional connectivity analyses provide information on the statistical relationships between brain region activations averaged over the entire scanning period, and it is possible that the reduced functional connectivity observed in our study reflects irregular activation patterns and desynchronized neuronal activity rather than loss of activation per se. Preclinical evidence suggests that intracellular inclusion of pathological proteins can cause aberrant cellular activity.^89^, ^90^ Consequently, Lewy body pathology within specific inhibitory interneurons in the amygdala^91^ could disrupt the homeostatic regulation of excitation and inhibition, which may manifest as decreased or inconsistent long‐range functional connectivity.^92^ This may cause episodic fluctuations in sensory integration and network stability, ultimately contributing to hallucinatory experiences. Incorporating behavioral paradigms that act as surrogates for visual hallucinatory phenomena,^93^, ^94^ in combination with dynamic fMRI approaches, could help identify state‐dependent changes that underlie abnormal perception in PD.

This study has several limitations that warrant consideration. First, the amygdala is a complex and heterogeneous structure composed of distinct nuclei with dissociable anatomical and functional connections.^30^, ^31^, ^55^, ^79^ However, considering the spatial resolution of our neuroimaging data, we opted to evaluate the amygdala as a whole, which may have obscured subregion‐specific contributions to VHs. Similarly, the NBM is challenging to localize with fMRI given its poorly defined anatomical boundaries.^95^ To address this, we used a probabilistic atlas to delineate the NBM, consistent with previous studies.^41^, ^96^, ^97^ Future studies using multiecho and high‐resolution imaging techniques may provide greater precision in examining functional connectivity of the basal forebrain and amygdalar subregions.^64^, ^98^ Second, although we demonstrated a mediating effect of NBM functional connectivity, the correlational nature of traditional functional connectivity analyses precludes definitive conclusions about causality. Future work using pharmacological manipulation or effective connectivity approaches such as dynamic causal modeling could clarify the directionality of influence between the NBM, amygdala, and attentional networks, and provide stronger mechanistic support for potential modulatory or hierarchical relationships. Third, both amygdala^55^ and cholinergic dysfunction^99^ have been linked to anxio‐depressive symptoms, sleep disturbances, and cognitive deficits, all of which have strong associations with VHs.^100^, ^101^, ^102^ Additional analyses controlling for anxiety, depression, and antidepressant use yielded largely consistent results. Nonetheless, the observed functional differences may reflect shared underlying pathology characteristic of a more severe or distinct nonmotor phenotype. Although general cognitive performance did not differ significantly between groups, both MMSE and MoCA are coarse screening tools, limiting our ability to examine associations between our findings and domain‐specific impairments relevant to VHs.^103^ Larger and more stratified samples may help to disentangle the contributions of overlapping or comorbid clinical features. Finally, because our study focused specifically on VHs, these findings may not generalize to other psychotic symptoms, such as delusions or hallucinations in other modalities.

In conclusion, our findings highlight the functional contributions of both NBM and amygdala to mechanisms underlying VHs in PD, providing new insights into how disruptions in subcortical modulation might participate in impaired sensory and attentional integration. Importantly, this work advances prior pathological and imaging research that has explored the relevance of these nuclei in isolation, showing that although changes within these regions may be necessary, taken alone they are not sufficient to fully account for VHs. Rather, our results emphasize the need for a more nuanced consideration of the interaction between multiple pathologically affected circuits. Although our focus was centered around the intersection of amygdala and cholinergic dysfunction, future research could extend these findings by examining how alterations across distributed cortical and subcortical systems converge to produce this complex and troubling symptom.

## Author Roles

(1) Research Project: A. Conception, B. Organization, C. Execution;

(2) Statistical Analysis: A. Design, B. Execution, C. Review and Critique;

(3) Manuscript Preparation: A. Writing of the First Draft, B. Review and Critique.

A.I.: 1A, 1B, 1C, 2A, 2B, 3A

L.C.: 1B, 1C, 2B, 2C, 3B

J.A.: 1C, 2C, 3B

A.K.: 1C, 2C, 3B

C.O.: 2C, 3B

S.J.G.L.: 1A, 2C, 3B

E.M.: 1A, 1B, 2A, 2C, 3B

## Full Financial Disclosures for All Authors (for the Previous 12 Months)

A.I. is a recipient of the University of Sydney Postgraduate Award scholarship. E.M. is supported by a National Health and Medical Research Council Emerging Leadership Fellowship (2008565) and the US Department of Defense Congressionally Directed Medical Research Program Early Investigator Grant (PD220061). S.J.G.L. is supported by a National Health and Medical Research Council Leadership Fellowship (1195830) and has received research funding from The Michael J. Fox Foundation and the Australian Research Council.

## Supporting information


**Table S1.** Missing cognitive data.
**Table S2.** Cortical networks at the regional level showing reduced amygdala and nucleus basalis of Meynert connectivity in Parkinson's disease patients with visual hallucinations compared to those without.
**Table S3.** Sensitivity model comparing between group functional connectivity including age, sex, HADS total scores and antidepressant use as covariates.
**Table S4.** Spearman's rank correlation analysis of cognitive, affective and sleep symptom scores with functional connectivity.
**Table S5.** Comparison of estimated amygdala and nucleus basalis of Meynert gray matter volumes.
**Table S6.** Seed to network analysis with primary auditory cortex as a control region.

## Data Availability

The data that support the findings of this study are available from the corresponding author upon reasonable request.
