# Supplementary material for: Combined Dysfunction of the Amygdala and Nucleus Basalis Underlies Visual Hallucinations in Parkinson's Disease
Source: Mov Disord. 2025 Aug 13;40(11):2381–92. doi: 10.1002/mds.70011 (PMC12661639; doi:10.1002/mds.70011)
Supplement: Supplementary file 1 — Table S1. Missing cognitive data. Table S2. Cortical networks at the regional level showing reduced amygdala and nucleus basalis of Meynert connectivity in Parkinson's disease patients with visual hallucinations compared to those without. Table S3. Sensitivity model comparing between group functional connectivity including age, sex, HADS total scores and antidepressant use as covariates. Table S4. Spearman's rank correlation analysis of cognitive, affective and sleep symptom scores with functional connectivity. Table S5. Comparison of estimated amygdala and nucleus basalis of Meynert gray matter volumes. Table S6. Seed to network analysis with primary auditory cortex as a control region. [file MDS-40-2381-s001.docx]

**Combined dysfunction of the amygdala and nucleus basalis underlies visual hallucinations in Parkinson’s disease**

**Supplementary material**

*Table of Contents*

[Functional imaging preprocessing and denoising 2](#_Toc202944597)

[Structural imaging preprocessing and volumetric analysis 3](#_Toc202944598)

[Mediation analysis 3](#_Toc202944599)

[Supplementary Table 1. Missing cognitive data 5](#_Toc202944600)

[Supplementary Table 2. Cortical networks at the regional level showing reduced amygdala and nucleus basalis of Meynert connectivity in Parkinson's disease patients with visual hallucinations compared to those without. 7](#_Toc202944601)

[Supplementary Table 3. Sensitivity model comparing between group functional connectivity including age, sex, HADS total scores and antidepressant use as covariates 10](#_Toc202944602)

[Supplementary Table 4. Spearman’s rank correlation analysis of cognitive, affective and sleep symptom scores with functional connectivity 10](#_Toc202944603)

[Supplementary Table 5. Comparison of estimated amygdala and nucleus basalis of Meynert grey matter volumes 10](#_Toc202944604)

[Supplementary Table 6. Seed to network analysis with primary auditory cortex as a control region 11](#_Toc202944605)

[References 12](#_Toc202944606)

# **Functional imaging preprocessing and denoising**

The functional connectivity results included in this manuscript come from preprocessing performed using fMRIPrep 21.0.2 ^1,2^; (RRID:SCR_016216), which is based on Nipype 1.8.1 ^3,4^; (RRID:SCR_002502).

The T1-weighted (T1w) image was corrected for intensity non-uniformity (INU) using N4BiasFieldCorrection ^5^; distributed with ANTs 2.3.1, (RRID:SCR_004757) and used as the T1w reference. The T1w reference was skull-stripped using the antsBrainExtraction.sh workflow (ANTs), with OASIS30ANTs as the target template. Brain tissue segmentation (CSF, WM, GM) was performed using fast ^6^; FSL 6.0.3, (RRID:SCR_002823). Spatial normalization to MNI152NLin6Asym and MNI152NLin2009cAsym templates was conducted using antsRegistration (ANTs 2.3.1) on brain-extracted images. The following templates were used: FSL's MNI ICBM 152 non-linear 6th Generation Asymmetric Average Brain Stereotaxic Registration Model ^7^; (RRID:SCR_002823) and ICBM 152 Nonlinear Asymmetrical template version 2009c ^8^; (RRID:SCR_008796).

A reference volume and its skull-stripped version were generated using a custom fMRIPrep methodology. Head-motion parameters were estimated using mcflirt (FSL 6.0.3). Slice-time correction was applied using 3dTshift (AFNI 20170202). The preprocessed BOLD time series were resampled to native space, followed by co-registration to the T1w reference using mri_coreg (FreeSurfer) and flirt (FSL).

Confounding time-series (e.g., FD, DVARS, global signals) were calculated, and CompCor regressors were extracted ^9^. BOLD runs were normalized to MNI152NLin6Asym and MNI152NLin2009cAsym spaces via antsApplyTransforms (ANTs) and mri_vol2surf (FreeSurfer).

Many operations used Nilearn 0.9.1 ^10^; (RRID:SCR_001362). For further details, please see [fMRIPrep's documentation](https://fmriprep.readthedocs.io/en/latest/workflows.html).

Following preprocessing, resting-state fMRI data were processed using Python and Nilearn to extract region-wise time-series data. Confounds, including motion parameters and WM/CSF signals, were regressed out using Nilearn’s load_confounds function, with motion and "basic" WM/CSF strategies. A custom parcellation atlas, provided as a NIfTI image, was used to define regions of interest (ROIs). The NiftiLabelsMasker from Nilearn was applied to the preprocessed BOLD images to extract average time-series data for each ROI. The masker included temporal filtering with a high-pass band filter (0.01 Hz) to remove low-frequency drifts and a low-pass band filter (0.1 Hz) to exclude high-frequency physiological noise. Data were extracted at a resolution of 2 mm in the MNI152NLin6Asym space. More information can be found at [fMRIDenoise's documentation](https://fmridenoise.readthedocs.io/).

# **Structural imaging preprocessing and volumetric analysis**

Anatomical data preprocessing was performed according to the standard CAT12 pipeline ^11^ which included denoising using a spatially adaptive non-local means filter, bias field inhomogeneity correction, and segmentation of the brain into grey matter, white matter, and cerebrospinal fluid. Spatial normalization to standard MNI template (MNI152NLin2009cAsym) was performed using the Diffeomorphic Anatomic Registration Through Exponentiated Lie algebra (DARTEL) algorithm. Preprocessing and segmentation accuracy were confirmed through visual inspection, supported by automated quality control metrics. The weighted interquartile range, which incorporates factors such as noise, intensity inhomogeneity, and image resolution was above 80% for all participants, indicating acceptable quality of anatomical data.

Grey matter volumes for the amygdala and nucleus basalis of Meynert (NBM) were extracted in the subject’s native space using the *Estimate mean values in ROI function* in CAT12. Estimated volumes were calculated by integrating voxel-wise grey matter density values within atlas-based ROIs defined by maximum probability maps. For each voxel within an ROI, grey matter density was multiplied by the weighting contained within the probabilistic map and summed across the ROI. Non-linear inverse deformation matrices generated during spatial normalization were used to warp atlases from the standard template space to the individual subject’s native space to check registration.

# **Mediation analysis**

Mediation analyses were performed to explore whether nucleus NBM functional connectivity with the ventral attentional network (VAN) mediated the association between amygdala-attentional network functional connectivity and the likelihood of a patient being classified as a hallucinator. Mediation analyses involve examining a series of relationships to determine how an intermediate (mediator) variable explains the connection between an independent variable and an outcome variable. Path *a* assesses the linear relationship between the independent variable (i.e. amygdala connectivity) and the mediating variable (i.e. NBM connectivity), while path *b* examines the association between the mediating variable and the outcome variable (i.e. presence of hallucinations). The indirect effect, representing the mediating role of NBM connectivity, is derived from the product of path *a* and path *b*. The total effect quantifies the combined influence of the independent and mediating variables on the outcome variable (path *c*), whereas the direct effect measures the relationship between the independent variable and the outcome variable while controlling for the mediating variable (path *c’*). An indirect effect is considered to mediate the total effect when the direct effect is no longer significant. Conversely, the indirect effect only partially mediates the total effect if the direct effect remains significant.

The mediation model incorporated both linear and logistic regression to account for continuous and binary outcomes, with age and sex included as covariates of no interest. Functional connectivity metrics were standardized prior to analysis to ensure comparability of regression coefficients. To validate the robustness of the mediation analyses, area under the receiver operating characteristic curve was calculated for each logistic regression model, revealing acceptable discriminative ability (~0.7) in predicting the likelihood of VH.

# **Supplementary Table 1. Missing cognitive data**

|  | **Number of missing data (%)** | |
| --- | --- | --- |
| **Cognitive Measure** | **PD-NoVH** | **PD-VH** |
| MoCA | 2/30 (6.7%) | 4/40 (10%) |

**List of full parcel names for the Yeo 17 network parcellation scheme^12^**

| **Abbreviation** | **Full parcel Name** |
| --- | --- |
| AntTemp | anterior temporal |
| Aud | auditory |
| Cent | central |
| Cinga | cingulate anterior |
| Cingm | mid-cingulate |
| Cingp | cingulate posterior |
| ExStr | extrastriate cortex |
| ExStrInf | extra-striate inferior |
| ExStrSup | extra-striate superior |
| FEF | frontal eye fields |
| FPole | frontal pole |
| FrMed | frontal medial |
| FrOper | frontal operculum |
| IFG | inferior frontal gyrus |
| Ins | insula |
| IPL | inferior parietal lobule |
| IPS | intraparietal sulcus |
| OFC | orbital frontal cortex |
| ParMed | parietal medial |
| ParOcc | parietal occipital |
| ParOper | parietal operculum |
| pCun | precuneus |
| pCunPCC | precuneus posterior cingulate cortex |
| PFCd | dorsal prefrontal cortex |
| PFCl | lateral prefrontal cortex |
| PFCld | lateral dorsal prefrontal cortex |
| PFClv | lateral ventral prefrontal cortex |
| PFCm | medial prefrontal cortex |
| PFCmp | medial posterior prefrontal cortex |
| PFCv | ventral prefrontal cortex |
| PHC | parahippocampal cortex |
| PostC | post central |
| PrC | precentral |
| PrCd | precentral dorsal |
| PrCv | precentral ventral |
| RSC | retrosplenial cortex |
| Rsp | retrosplenial |
| S2 | S2 |
| SPL | superior parietal lobule |
| ST | superior temporal |
| Striate | striate cortex |
| StriCal | striate calcarine |
| Temp | temporal |
| TempOcc | temporal occipital |
| TempPar | temporal parietal |
| TempPole | temporal pole |

| **Supplementary Table 2. Cortical networks at the regional level showing reduced amygdala and nucleus basalis of Meynert connectivity in Parkinson's disease patients with visual hallucinations compared to those without.** | | | | | |
| --- | --- | --- | --- | --- | --- |
| **Left amygdala – Visual Network functional connectivity** | | | | | |
| **ROI name** | **MNI coordinates (mm)** | | | ***p*-value** | **Cohen’s *d*** |
|  | **x** | **y** | **z** |  |  |
| LHVisCentExStr1 | -36 | -62 | -17 | 0.011 | -0.628 |
| LHVisCentExStr2 | -23 | -73 | -10 | 0.014 | -0.613 |
| LHVisCentExStr11 | -25 | -85 | 21 | 0.035 | -0.517 |
| LHVisCentExStr6 | -41 | -87 | -3 | 0.014 | -0.591 |
| LHVisCentExStr8 | -24 | -96 | 6 | 0.030 | -0.527 |
| LHVisPeriExStrInf1 | -24 | -55 | -8 | 0.001 | -0.864 |
| LHVisPeriExStrInf3 | -7 | -76 | -6 | 0.007 | -0.674 |
| LHVisPeriExStrInf4 | -13 | -43 | -5 | 0.010 | -0.638 |
| LHVisPeriExStrInf5 | -14 | -57 | 1 | 0.018 | -0.579 |
| LHVisPeriStriCal1 | -5 | -88 | 2 | 0.017 | -0.604 |
| LHVisPeriStriCal2 | -7 | -74 | 9 | 0.017 | -0.587 |
| RHVisCentExStr1 | 36 | -53 | -17 | 0.009 | -0.635 |
| RHVisCentExStr3 | 23 | -74 | -11 | 0.003 | -0.739 |
| RHVisCentExStr7 | 35 | -89 | 2 | 0.010 | -0.639 |
| RHVisCentStriate1 | 8 | -92 | -2 | 0.047 | -0.485 |
| RHVisPeriExStrInf1 | 26 | -52 | -9 | 0.027 | -0.552 |
| RHVisPeriExStrInf3 | 9 | -72 | -5 | 0.013 | -0.593 |
| RHVisPeriExStrInf5 | 18 | -45 | -3 | 0.018 | -0.588 |
| RHVisPeriExStrSup2 | 5 | -80 | 24 | 0.014 | -0.601 |

| **Right amygdala – Visual Network functional connectivity** | | | | | |
| --- | --- | --- | --- | --- | --- |
| **ROI name** | **MNI coordinates (mm)** | | | ***p*-value** | **Cohen’s *d*** |
|  | **x** | **y** | **z** |  |  |
| LHVisCentExStr1 | -36 | -62 | -17 | 0.0415 | -0.502 |
| LHVisCentExStr2 | -23 | -73 | -10 | 0.040 | -0.503 |
| LHVisCentExStr11 | -25 | -85 | 21 | 0.036 | -0.512 |
| LHVisCentExStr6 | -41 | -87 | -3 | 0.021 | -0.566 |
| LHVisCentExStr8 | -24 | -96 | 6 | 0.008 | -0.668 |
| LHVisPeriExStrInf1 | -24 | -55 | -8 | 0.003 | -0.753 |
| LHVisPeriExStrInf3 | -7 | -76 | -6 | 0.045 | -0.491 |
| LHVisPeriExStrInf4 | -13 | -43 | -5 | 0.006 | -0.683 |
| LHVisPeriExStrInf5 | -14 | -57 | 1 | 0.028 | -0.547 |
| LHVisPeriExStrSup1 | -19 | -65 | 7 | 0.047 | -0.490 |
| LHVisPeriExStrSup3 | -12 | -71 | 20 | 0.042 | -0.500 |
| LHVisPeriStriCal1 | -5 | -88 | 2 | 0.028 | -0.536 |
| LHVisPeriStriCal2 | -7 | -74 | 9 | 0.023 | -0.559 |
| RHVisPeriExStrInf1 | 26 | -52 | -9 | 0.020 | -0.572 |
| RHVisPeriExStrInf3 | 9 | -72 | -5 | 0.045 | -0.492 |

| **Left amygdala – Dorsal Attentional Network functional connectivity** | | | | | | | |
| --- | --- | --- | --- | --- | --- | --- | --- |
| **ROI name** | **MNI coordinates (mm)** | | | | | ***p*-value** | **Cohen’s *d*** |
|  | **x** | **y** | | **z** | |  |  |
| LH_DorsAttnA_SPL_1 | -26 | | -70 | | 31 | 0.015 | -0.607 |
| LH_DorsAttnA_TempOcc_2 | -33 | | -42 | | -21 | 0.007 | 0.676 |
| LH_DorsAttnA_TempOcc_4 | -45 | | -70 | | -8 | 0.009 | -0.646 |
| LH_DorsAttnB_PostC_1 | -61 | | -23 | | 33 | 0.041 | -0.507 |
| LH_DorsAttnB_PostC_2 | -55 | | -20 | | 41 | 0.029 | -0.538 |
| LH_DorsAttnB_PostC_3 | -55 | | -32 | | 45 | 0.014 | -0.617 |
| LH_DorsAttnB_PostC_4 | -46 | | -29 | | 44 | 0.008 | -0.666 |
| LH_DorsAttnB_PostC_5 | -39 | | -37 | | 49 | 0.033 | -0.536 |
| RH_DorsAttnA_ParOcc_1 | 48 | | -66 | | 4 | 0.011 | -0.629 |
| RH_DorsAttnA_ParOcc_3 | 36 | | -79 | | 24 | 0.041 | -0.507 |
| RH_DorsAttnA_SPL_1 | 29 | | -78 | | 37 | 0.006 | -0.673 |
| RH_DorsAttnA_SPL_4 | 31 | | -64 | | 53 | 0.041 | -0.508 |
| RH_DorsAttnA_TempOcc_2 | 50 | | -49 | | -18 | 0.044 | -0.494 |
| RH_DorsAttnB_PostC_1 | 61 | | -14 | | 30 | 0.005 | -0.713 |
| RH_DorsAttnB_PostC_5 | 35 | | -36 | | 51 | 0.015 | -0.607 |
| RH_DorsAttnB_PostC_7 | 24 | | -50 | | 68 | 0.032 | -0.527 |
| RH_DorsAttnB_TempOcc_1 | 34 | | -37 | | -23 | 0.033 | -0.517 |

| **Left amygdala – Ventral Attentional Network functional connectivity** | | | | | |  |
| --- | --- | --- | --- | --- | --- | --- |
| **ROI name** | **MNI coordinates (mm)** | | | ***p*-value** | **Cohen’s *d*** |  |
|  | **x** | **y** | **z** |  |  |  |
| LH_SalVentAttnA_FrMed_1 | -7 | 0 | 41 | 0.021 | -0.563 |  |
| LH_SalVentAttnA_FrMed_2 | -5 | 9 | 48 | 0.015 | -0.594 |  |
| LH_SalVentAttnA_FrMed_3 | -8 | -3 | 71 | 0.006 | -0.694 |  |
| LH_SalVentAttnA_FrOper_1 | -50 | 1 | 5 | 0.040 | -0.506 |  |
| LH_SalVentAttnA_ParOper_2 | -58 | -44 | 27 | 0.033 | -0.522 |  |
| LH_SalVentAttnA_ParOper_3 | -61 | -36 | 33 | 0.015 | -0.595 |  |
| LH_SalVentAttnB_PFCl_2 | -29 | 43 | 30 | 0.015 | -0.595 |  |
| RH_SalVentAttnA_FrMed_1 | 7 | 2 | 43 | 0.013 | -0.606 |  |
| RH_SalVentAttnA_FrMed_4 | 16 | 7 | 69 | 0.042 | -0.500 |  |
| RH_SalVentAttnA_FrOper_2 | 49 | 5 | 3 | 0.037 | -0.521 |  |
| RH_SalVentAttnA_Ins_3 | 40 | -10 | -4 | 0.008 | -0.674 |  |
| RH_SalVentAttnA_ParMed_1 | 11 | -17 | 41 | 0.021 | -0.567 |  |
| RH_SalVentAttnA_ParMed_3 | 10 | -43 | 53 | 0.019 | -0.583 |  |
| RH_SalVentAttnA_ParOper_1 | 58 | -31 | 24 | 0.001 | -0.836 |  |
| RH_SalVentAttnA_ParOper_2 | 60 | -22 | 22 | 0.031 | -0.537 |  |
| RH_SalVentAttnA_ParOper_3 | 63 | -26 | 38 | 0.018 | -0.587 |  |
| RH_SalVentAttnA_PrC_1 | 51 | 3 | 41 | 0.033 | -0.525 |  |
| RH_SalVentAttnB_IPL_1 | 62 | -37 | 37 | 0.006 | -0.680 |  |
| RH_SalVentAttnB_PFCl_1 | 42 | 46 | 14 | 0.001 | -0.857 |  |
| **Left nucleus basalis of Meynert – Ventral Attentional Network functional connectivity** | | | | | | |
| **ROI name** | | **MNI coordinates (mm)** | | | ***p*-value** | **Cohen’s *d*** |
|  |  | **x** | **y** | **z** |  |  |
| LH_SalVentAttnA_FrOper_1 | | -50 | 1 | 5 | 0.035 | -0.519 |
| RH_SalVentAttnA_FrOper_2 | | 49 | 5 | 3 | 0.015 | -0.616 |
| RH_SalVentAttnA_Ins_2 | | 41 | 8 | -3 | 0.047 | -0.485 |
| RH_SalVentAttnA_ParOper_1 | | 58 | -31 | 24 | 0.008 | -0.656 |
| RH_SalVentAttnB_PFCl_1 | | 42 | 46 | 14 | 0.004 | -0.725 |
| RH_SalVentAttnB_PFCl_2 | | 25 | 54 | 25 | 0.012 | -0.610 |
| RH_SalVentAttnB_PFClv_1 | | 49 | 40 | 5 | 0.049 | -0.482 |
| RH_SalVentAttnB_PFCmp_1 | | 8 | 35 | 25 | 0.018 | -0.590 |

Region labels follow the Schaefer-400 17-network 2mm parcellation labels

# **Supplementary Table 3. Sensitivity model comparing between group functional connectivity including age, sex, HADS total scores and antidepressant use as covariates**

| **Seed-network FC** | ***p* uncorrected** | ***p* FDR** |
| --- | --- | --- |
| Left Amygdala-VIS | <0.001 | 0.008 |
| Right Amygdala-VIS | 0.045 | 0.122 |
| Left Amygdala-DAN | 0.007 | 0.046 |
| Left Amygdala-VAN | 0.004 | 0.044 |
| Left NBM-VAN | 0.021 | 0.082 |

Dorsal Attentional Network = DAN; Functional connectivity = FC; Nucleus Basalis of Meynert = NBM; Ventral Attentional Network = VAN; Visual Network = VIS

# **Supplementary Table 4. Spearman’s rank correlation analysis of cognitive, affective and sleep symptom scores with functional connectivity**

| **Seed-network FC** | **HADS Anxiety** | **HADS Depression** | **RBDSQ** | **MoCA** |
| --- | --- | --- | --- | --- |
| Left Amygdala-VIS | rho = 0.047  *p* = 0.798 | rho = 0.045  *p* = 0.809 | rho = 0.142  *p* = 0.466 | rho = 0.258  *p* = 0.168 |
| Right Amygdala-VIS | rho = -0.197  *p* = 0.301 | rho = -0.087  *p* = 0.650 | rho = -0.065  *p* = 0.728 | rho = 0.260  *p* = 0.165 |
| Left Amygdala-DAN | rho = -0.340  *p* = 0.068 | rho = -0.209  *p* = 0.286 | rho = 0.041  *p* = 0.831 | rho = 0.118  *p* = 0.536 |
| Left Amygdala-VAN | rho = 0.060  *p* = 0.743 | rho = 0.129  *p* = 0.506 | rho = -0.196  *p* = 0.299 | rho = -0.125  *p* = 0.510 |
| Left NBM-VAN | rho = -0.020  *p* = 0.920 | rho = 0.012  *p* = 0.949 | rho = -0.104  *p* = 0.583 | rho = -0.064  p = 0.738 |

Dorsal Attentional Network = DAN; Functional connectivity = FC; Hospital Anxiety and Depression scale = HADS; Montreal Cognitive Assessment = MoCA; Nucleus Basalis of Meynert = NBM; Rapid Eye Movement Screening questionnaire = RBDSQ; Ventral Attentional Network = VAN; Visual Network = VIS

# **Supplementary Table 5. Comparison of estimated amygdala and nucleus basalis of Meynert grey matter volumes**

| **Region of interest** | **PD-NoVH** | **PD-VH** | ***p* FDR** |
| --- | --- | --- | --- |
| Left Amygdala | 1.692 ± 0.16 mL | 1.674 ± 0.12 mL | 0.711 |
| Right Amygdala | 1.697 ± 0.16 mL | 1.652 ± 0.10 mL | 0.279 |
| Left NBM | 0.152 ± 0.02 mL | 0.143 ± 0.02 mL | 0.106 |
| Right NBM | 0.110 ± 0.01 mL | 0.101 ± 0.01 mL | 0.279 |

All volumes are adjusted for estimated total intracranial volume

# **Supplementary Table 6. Seed to network analysis with primary auditory cortex as a control region**

|  | **Left seed** | | **Right seed** | |
| --- | --- | --- | --- | --- |
| **Seed-network FC** | ***p* FDR** | **Cohen’s *d*** | ***p* FDR** | **Cohen’s *d*** |
| A1-VIS | 0.072 | -0.696 | 0.246 | -0.372 |
| A1-DAN | 0.233 | -0.444 | 0.233 | -0.447 |
| A1-VAN | 0.092 | -0.631 | 0.072 | -0.627 |
| A1-FPN | 0.233 | -0.485 | 0.253 | -0.408 |
| A1-DMN | 0.246 | -0.491 | 0.301 | -0.464 |

Post-hoc, exploratory control analysis was performed to evaluate the anatomical specificity of our findings. Primary auditory cortex (A1) was chosen as a control seed as it is unlikely to associate with hallucinations in the visual modality. A1 was defined using the Schaefer-400 2mm parcellation (MNI coordinates; left: x = -50, y = -9, z = 0; right: x = 53, y = 3, z = -6). Between group comparisons were controlled for age, sex, anxiety and depressions scores (HADS) and antidepressant use. Default Mode Network = DMN; Dorsal Attention Network = DAN; Frontoparietal Network = FPN; Ventral Attention Network = VAN; Visual Network = VIS

# **References**

1. Esteban O, Ciric R, Finc K, et al. Analysis of task-based functional MRI data preprocessed with fMRIPrep. *Nature Protocols*. 2020/07/01 2020;15(7):2186-2202. doi:10.1038/s41596-020-0327-3

2. Esteban O, Markiewicz CJ, Blair RW, et al. fMRIPrep: a robust preprocessing pipeline for functional MRI. *Nature methods*. 2019;16(1):111-116.

3. Gorgolewski K, Burns CD, Madison C, et al. Nipype: A Flexible, Lightweight and Extensible Neuroimaging Data Processing Framework in Python. Original Research. *Frontiers in Neuroinformatics*. 2011-August-22 2011;5doi:10.3389/fninf.2011.00013

4. Gorgolewski KJ, Durnez J, Poldrack RA. Preprocessed consortium for neuropsychiatric phenomics dataset. *F1000Research*. 2017;6

5. Tustison NJ, Avants BB, Cook PA, et al. N4ITK: improved N3 bias correction. *IEEE transactions on medical imaging*. 2010;29(6):1310-1320.

6. Zhang Y, Brady M, Smith S. Segmentation of brain MR images through a hidden Markov random field model and the expectation-maximization algorithm. *IEEE transactions on medical imaging*. 2001;20(1):45-57.

7. Evans AC, Janke AL, Collins DL, Baillet S. Brain templates and atlases. *NeuroImage*. 2012/08/15/ 2012;62(2):911-922. doi:<https://doi.org/10.1016/j.neuroimage.2012.01.024>

8. Fonov VS, Evans AC, McKinstry RC, Almli CR, Collins D. Unbiased nonlinear average age-appropriate brain templates from birth to adulthood. *NeuroImage*. 2009;47:S102.

9. Behzadi Y, Restom K, Liau J, Liu TT. A component based noise correction method (CompCor) for BOLD and perfusion based fMRI. *Neuroimage*. 2007;37(1):90-101.

10. Abraham A, Pedregosa F, Eickenberg M, et al. Machine learning for neuroimaging with scikit-learn. *Frontiers in neuroinformatics*. 2014;8:71792.

11. Gaser C, Dahnke R, Thompson PM, Kurth F, Luders E, the Alzheimer's Disease Neuroimaging Initiative. CAT: a computational anatomy toolbox for the analysis of structural MRI data. *GigaScience*. 2024;13:giae049. doi:10.1093/gigascience/giae049

12. Yeo BT, Krienen FM, Sepulcre J, et al. The organization of the human cerebral cortex estimated by intrinsic functional connectivity. *J Neurophysiol*. Sep 2011;106(3):1125-65. doi:10.1152/jn.00338.2011
